# A multi-site cutting device implements efficiently the divide-and-conquer strategy in tumor sampling

**DOI:** 10.12688/f1000research.9091.2

**Published:** 2016-07-26

**Authors:** Jose I. Lopez, Jesus M. Cortes

**Affiliations:** 1 Department of Pathology, Cruces University Hospital, Barakaldo, Spain; 2 Biomarkers in Cancer Unit, Biocruces Research Institute, Barakaldo, Spain; 3 University of the Basque Country, Leioa, Spain; 4 Quantitative Biomedicine Unit, Biocruces Research Institute, Barakaldo, Spain; 5 Ikerbasque: The Basque Foundation for Science, Bilbao, Spain; 6 Department of Cell Biology and Histology, University of the Basque Country, Leioa, Spain

**Keywords:** Tumor sampling, cutting grid, divide and conquer, clear cell renal cell carcinoma, intratumor heterogeneity, pathology routine

## Abstract

We recently showed that in order to detect intra-tumor heterogeneity a Divide-and-Conquer (DAC) strategy of tumor sampling outperforms current routine protocols. This paper is a continuation of this work, but here we focus on DAC implementation in the Pathology Laboratory. In particular, we describe a new simple method that makes use of a cutting grid device and is applied to clear cell renal cell carcinomas for DAC implementation. This method assures a thorough sampling of large surgical specimens, facilitates the demonstration of intratumor heterogeneity, and saves time to pathologists in the daily practice. The method involves the following steps: 1. Thin slicing of the tumor (by hand or machine), 2. Application of a cutting grid to the slices ( *e.g*., a French fry cutter), resulting in multiple tissue cubes with fixed position within the slice, 3. Selection of tissue cubes for analysis, and finally, 4. Inclusion of selected cubes into a cassette for histological processing (with about eight tissue fragments within each cassette). Thus, using our approach in a 10 cm in-diameter-tumor we generate 80 tumor tissue fragments placed in 10 cassettes and, notably, in a tenth of time. Eighty samples obtained across all the regions of the tumor will assure a much higher performance in detecting intratumor heterogeneity, as proved recently with synthetic data.

## Introduction

In the light of current findings provided by numerous sequencing tools, it is known that practically all human neoplasms display some degree of intratumor heterogeneity (ITH) ^1^. Characteristically, ITH is not uniformly distributed along the tumor; instead, it shows a regional distribution following a stochastic pattern, the final result being unique, unpredictable, and dynamically varying along the time ^2^. The correct identification of ITH is mandatory now that targeted therapies are offering promising results to patients ^3^, but pathologists - the specialists in charge of tumor selection for analysis - seem to have not given, so far, an appropriate answer to this issue.

We have recently proposed a reliable, affordable and time-saving solution to this problem ^4^. The goal is twofold; to improve ITH detection and to perform ITH at affordable laboratory costs. This simple solution is based on the divide-and-conquer algorithm (DAC). Noteworthy, tumor sampling following DAC outperforms the routine protocol sampling for identifying ITH and, it does it, at a similar cost ^4^. However, pathologists could consider that DAC is a time-consuming method when grossing, which might make it difficult to introduce it in routine practice. In this brief report, we describe a simple procedure to overcome this problem.

## Method and results

The so-called DAC algorithm ^5^ is based on recursively breaking down a problem in smaller parts (divide) until these are simple enough to be solved directly (conquer). Then, partial solutions are combined to solve the original problem. DAC strategies have been largely applied in science to solve complex problems, including several challenging issues in biomedical areas. As early as in 1967, DAC helped clinicians to correlate hypoglycemia with infantile convulsions ^6^. In addition, DAC has been useful in cell biology and oncology, for instance in selecting the appropriate cells for biological experiments ^7^ and, more recently, in helping to decipher breast cancer heterogeneity ^8^.

**Figure 1.**
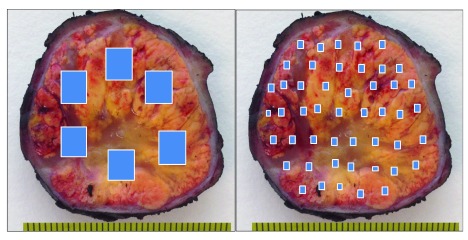
Schematic representation of routine (left) and divide-and-conquer (right) strategies in tumor sampling.

Here DAC is applied to clear cell renal cell carcinomas (ccRCCs), since these tumors are frequently large and, for this reason, impossible to be totally sampled. Any other large tumor, however, can benefit from this method. The DAC strategy ( Figure 1) requires the pathologist to select, instead of a few large fragments, a substantial number of small ones widely distributed along the entire tumor. However, pathologists under a daily routine pressure can perceive this method as laborious and time-consuming.

**Figure 2.**
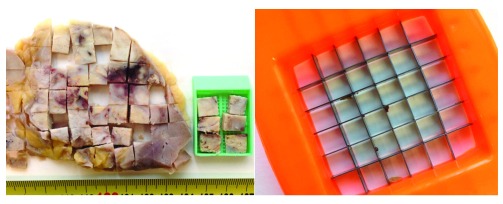
Tumor tissue sampling after being divided with a cutting grid.

A simple device consisting of a cutting grid (here, a potato cutter) will overcome this inconvenience. When applied directly to the whole tumor surface previously thin-sliced, the grid will cut it into small cubes in one shot ( Figure 2). Next, the pathologist’s decision will consist simply in selecting the cubes that will be processed for analysis as previously reported ^4^. The method can be applied (and has been tested) to both fresh and formalin-fixed tissue, saves time, and assures a uniform sampling distribution along the tumor. The objective for improving efficiency of targeted therapies is the discovery of the complete ITH spectrum, and not its exact location. Thus the selected cubes included in the cassettes (six to eight cubes per cassette) will provide much more thorough information of the tumor, both under the microscope as well as at the molecular level.

A big tumor size is not a limitation. If the tumor is bigger than the cutting grid, the pathologist may apply the DAC strategy and subdivide the whole slice into smaller parts before applying the cutting device to each one of these portions. Since the final goal of this strategy is a thorough tumor sampling, the slicing in the grossing room does not need to be necessarily perfect. Obtaining a whole and homogeneous slice in a huge tumor, in a tumor with heterogeneous consistency, or in a tumor with multifocal necrosis, may be a difficult task under daily routine pressure. For these cases, partial thin slices of the whole tumor circumference might be also appropriate for this sampling strategy. In fact, it is a more advantageous and time-saving attitude to obtain multiple thin (with thickness smaller than 3 mm) sectorial slices along the tumor than complete irregular thicker (bigger than 3 mm) slices, as the cubes obtained by the subsequent application of the cutting grid will not need to be additionally thinned one by one with a scalpel to fit them into the cassette.

We have designed the DAC strategy to enhance ITH detection in big tumors at the same cost ^4^. For this reason, we propose to respect what the international protocols advice, that is, one cassette per centimeter of tumor diameter. Extreme cases will follow the same rule. Thus, a 20 cm in diameter renal tumor would generate 20 cassettes harboring in total 120 to 160 tumor samples (six to eight per cassette) thus assuring a thorough sampling. As usual, the decision of which are the cubes to be selected in the sampling is a pathologist's personal decision. The common sense says that any area with different consistency or color should be sampled, but if the tumor is totally homogeneous by naked eye we propose a balanced geographical selection of cubes in each tumor slice. Additionally, a small fragment of normal renal tissue can be included in some cassettes to have an internal control for eventual immunohistochemical or molecular studies.

## Discussion

The use of the DAC method to help sampling strategies is not new. Indeed some authors have applied the algorithm in particle physics to improve the diffusion sampling in generalized ensemble simulations ^9^. This approach, or any other with scientific basis, has not been implemented for tissue selection in Pathology laboratories so far, since the pathologists did not consider tumor sampling a complex problem in the pre-molecular era.

An experience-based reasoning says that this option will save pathologist’s time when handling large tumors, in a manner which is inexpensive and reliable at the same time. In combination with the changes proposed for the technician training in our previous report ^4^, this new alternative will make the pathologists’ routine much faster and robust providing an integrated solution to fulfill basic researchers’ expectations ^10^. If the DAC strategy is adopted as a suitable method to increase the amount of information given to oncologists, pathologist's routine will move from the classic big-fragments-into-the-cassette routine to a sort of rudimentary tissue microarray building, as recently proposed ^4^.

Figures are demonstrative. For instance, the DAC strategy applied to a ccRCC of 10 cm in diameter - a quite common situation in routine pathology - will generate approximately 80 small samples (of about 4–5 mm in size) that would be included in 10 cassettes for a thorough tumor examination. Importantly to remark, the same 10 cm in diameter tumor would need also 10 cassettes for the analysis, with one tumor sample per cassette, in the case of routine sampling protocols ^11^.

Depending on the pathologist’s skills, the time to collect 80 small samples in the grossing room is variable, but in any case, long. For this reason, any successful alternative must necessarily overcome this hurdle. A feasible choice would be an electric bacon slicer, but a long bladed knife will also work. To note, slicing electric machines are being increasingly used in pathology for handling radical prostatectomies ^12^ and other surgical specimens ^13^, and they are the first step in the whole-mounting processing for tumor mapping. In this case, the obtained ccRCC slices can be quickly cut in one shot by pressing on the entire tumor surface with a cutting grid. The procedure will generate many cubes ready to be included within a cassette. If we assume that tumor sampling following a DAC strategy is appropriate for improving ITH detection, the use of a cutting grid will shorten significantly the total process ensuring a uniform and widespread selection of the samples. A straightforward estimation with some practical cases indicates that the time for obtaining 80 samples with this method is reduced to a tenth as we cut 10 small tumor pieces at the same time.

DAC strategy, by definition, is not necessary in small tumors if they can be totally sampled. It is the policy of any Laboratory to fix the maximum tumor size for performing a total tumor sampling, since there are no precise recommendations in the international protocols. In this setting, the final decision will probably depend on the routine pressure, local customs and habits and, more important, on the varying annual budget assigned to the Laboratory of Pathology.

## Conclusions

The present paper describes a new method for tumor sampling in routine pathology inspired by the DAC algorithm ^4^. Once DAC has been proved to be efficient for ITH detection, we expect that the use of a cutting grid will make affordable its widespread application. Objectives are twofold: ITH detection improvement and time optimization (cost) in Pathology laboratories.

## References

[1] Gay L Baker AM Graham TA: Tumour Cell Heterogeneity [version 1; referees: 5 approved]. *F1000Res.* 2016;5: pii: F1000 Faculty Rev-238. 10.12688/f1000research.7210.1 26973786 PMC4776671

[2] Gerlinger M Rowan AJ Horswell S: Intratumor heterogeneity and branched evolution revealed by multiregion sequencing. *N Engl J Med.* 2012;366(10):883–892. 10.1056/NEJMoa1113205 22397650 PMC4878653

[3] Hiley C de Bruin EC McGranahan N: Deciphering intratumor heterogeneity and temporal acquisition of driver events to refine precision medicine. *Genome Biol.* 2014;15(8):453. 10.1186/s13059-014-0453-8 25222836 PMC4281956

[4] López JI Cortés JM: A divide-and-conquer strategy in tumor sampling enhances detection of intratumor heterogeneity in routine pathology: A modeling approach in clear cell renal cell carcinoma [version 1; referees: 4 approved]. *F1000Res.* 2016;5:385. 10.12688/f1000research.8196.1 27127618 PMC4830216

[5] Cormen TH Leiserson CE Rivest RL: Introduction to Algorithms.2nd Edition, MIT Press,2001 Reference Source

[6] Divide and conquer. *JAMA.* 1967;202(13):1144. 10.1001/jama.1967.03130260066014 6072710

[7] Eisenstein M: Cell sorting: Divide and conquer. *Nature.* 2006;441(7097):1179–1185. 10.1038/4411179a 16810261

[8] Kristensen VN: Divide and conquer: the genetic basis of molecular subclassification of breast cancer. *EMBO Mol Med.* 2011;3(4):183–185. 10.1002/emmm.201100128 21394915 PMC3377072

[9] Min D Yang W: A divide-and-conquer strategy to improve diffusion sampling in generalized ensemble simulations. *J Chem Phys.* 2008;128(9):094106. 10.1063/1.2834500 18331086

[10] Soultati A Stares M Swanton C: How should clinicians address intratumour heterogeneity in clear cell renal cell carcinoma? *Curr Opin Urol.* 2015;25(5):358–366. 10.1097/MOU.0000000000000204 26125509

[11] Trpkov K Grignon DJ Bonsib SM: Handling and staging of renal cell carcinoma: the International Society of Urological Pathology Consensus (ISUP) conference recommendations. *Am J Surg Pathol.* 2013;37(10):1505–1517. 10.1097/PAS.0b013e31829a85d0 24025521

[12] Egevad L: Handling of radical prostatectomy specimens. *Histopathology.* 2012;60(1):118–124. 10.1111/j.1365-2559.2011.04002.x 22212081

[13] Helliwell TR: ACP Best Practice No 157. Guidelines for the laboratory handling of laryngectomy specimens. *J Clin Pathol.* 2000;53(3):171–176. 10.1136/jcp.53.3.171 10823133 PMC1731165

