# Supplementary material for: A multi-site cutting device implements efficiently the divide-and-conquer strategy in tumor sampling
Source: F1000Res. 2016 Jul 26;5:1587. Originally published 2016 Jul 6. [Version 2] doi: 10.12688/f1000research.9091.2 (PMC4965694; doi:10.12688/f1000research.9091.2)
Supplement: Peer review history

# Peer review: Referee response for version 2

**DOI:** 10.5256/f1000research.9998.r15228

**Published:** 2016-07-27

**Authors:** Thomas V. Colby

**Affiliations:** 1 Department of Laboratory Medicine and Pathology, Mayo Clinic, Scottsdale, AZ, USA

I have read this submission. I believe that I have an appropriate level of expertise to confirm that it is of an acceptable scientific standard.

# Peer review: Referee response for version 2

**DOI:** 10.5256/f1000research.9998.r15226

**Published:** 2016-07-27

**Authors:** Christopher DM Fletcher

**Affiliations:** 1 Department of Pathology, Brigham and Women's Hospital, Boston, MA, USA

The authors have responded constructively to the referees' comments and I believe that the paper is improved and more useful to readers as a consequence.

I have read this submission. I believe that I have an appropriate level of expertise to confirm that it is of an acceptable scientific standard.

# Peer review: Referee response for version 1

**DOI:** 10.5256/f1000research.9785.r15138

**Published:** 2016-07-21

**Authors:** Manuel Areias Sobrinho Simoes

**Affiliations:** 1 Institute of Molecular Pathology and Immunology, University of Porto, Porto, Portugal; 2 Department of Pathology and Oncology, University of Porto, Porto, Portugal

The article “A multi-site cutting device implements efficiently the divide-and-conquer strategy in tumor sampling” represents an intelligent and practical evolution of the previous article of the same authors on the challenging issues of intratumor heterogeneity and tumor sampling.

 Tumor heterogeneity is one of the most important features of any approach to oncobiology and/or oncology. Together with other factors with which it is related – topography and time (evolution) – tumor heterogeneity is a key issue of cancer diagnosis and cancer treatment.

 López and Cortes addressed this issue adequately in their previous article demonstrating the importance of finding ways of evaluating tumor heterogeneity and topography via a clever and innovative strategy – Divide-and conquer (DAC) strategy –  of tumor sampling.

 I must confess I liked very much the previous article but I did not know how much effort one would need to implement a successful DAC strategy. It was therefore an agreeable surprise to see the continuation of the work having the real life of a Pathology Laboratory as the objective.

 I think the authors describe reasonably well the method they have “invented” and I concur with them it looks a simple procedure that assures a rather complete sampling of large surgical specimens in a relatively short period of time. However, for the sake of utility, I think the authors should provide a more detailed description of the “technicalities” of the procedure.

I have read this submission. I believe that I have an appropriate level of expertise to confirm that it is of an acceptable scientific standard, however I have significant reservations, as outlined above.

# Peer review: Referee response for version 1

**DOI:** 10.5256/f1000research.9785.r14804

**Published:** 2016-07-08

**Authors:** Christopher DM Fletcher

**Affiliations:** 1 Department of Pathology, Brigham and Women's Hospital, Boston, MA, USA

This is valuable and innovative work, which provides a simple and practical approach to more efficient sampling of larger human tumors, with the goal of better determining tumor heterogeneity.

 There are some omissions in the description of the technique, which would make adoption of the technique easier for others. Specifically, the authors should state clearly the recommended thickness of the whole tumor slice, whether using a long-bladed knife or slicing machine. Clearly the slice thickness must fit in the tissue processing cassette - so probably less than 3 mm - do the authors think that 3 mm slices of a large mass will generally remain intact/not fall apart before being cut with the grid. The second, perhaps more important, point is the authors need to state clearly how much of a given tumor they believe should be sampled. In their 10 cm example, it seems like they would submit virtually the whole tumor but this is not clear. How much would they submit from a 20 cm tumor? Conversely, how big does a tumor need to be before this type of sampling technique is advised? Presumably smaller tumors (e.g. 5 cm) could be sampled in the conventional/routine fashion with larger pieces? Clarification of these points will enhance the value of the authors' proposal.

I have read this submission. I believe that I have an appropriate level of expertise to confirm that it is of an acceptable scientific standard, however I have significant reservations, as outlined above.

# Peer review: Referee response for version 1

**DOI:** 10.5256/f1000research.9785.r14803

**Published:** 2016-07-07

**Authors:** Thomas V. Colby

**Affiliations:** 1 Department of Laboratory Medicine and Pathology, Mayo Clinic, Scottsdale, AZ, USA

A simple and practical proposal for tumor sampling tissue. One suggestion: give some guidance as to which among multiple tissue cubes would be put in the cassettes.

I have read this submission. I believe that I have an appropriate level of expertise to confirm that it is of an acceptable scientific standard, however I have significant reservations, as outlined above.
